# Hepatitis E Virus Replication

**DOI:** 10.3390/v11080719

**Published:** 2019-08-06

**Authors:** Robert LeDesma, Ila Nimgaonkar, Alexander Ploss

**Affiliations:** Department of Molecular Biology, Lewis Thomas Laboratory, Princeton University, Princeton, NJ 08544, USA

**Keywords:** hepatitis E, hepatitis E virus, ORF1, viral replication

## Abstract

Hepatitis E virus (HEV) is a small quasi-enveloped, (+)-sense, single-stranded RNA virus belonging to the *Hepeviridae* family. There are at least 20 million HEV infections annually and 60,000 HEV-related deaths worldwide. HEV can cause up to 30% mortality in pregnant women and progress to liver cirrhosis in immunocompromised individuals and is, therefore, a greatly underestimated public health concern. Although a prophylactic vaccine for HEV has been developed, it is only licensed in China, and there is currently no effective, non-teratogenic treatment. HEV encodes three open reading frames (ORFs). ORF1 is the largest viral gene product, encoding the replicative machinery of the virus including a methyltransferase, RNA helicase, and an RNA-dependent RNA polymerase. ORF1 additionally contains a number of poorly understood domains including a hypervariable region, a putative protease, and the so-called ‘X’ and ‘Y’ domains. ORF2 is the viral capsid essential for formation of infectious particles and ORF3 is a small protein essential for viral release. In this review, we focus on the domains encoded by ORF1, which collectively mediate the virus’ asymmetric genome replication strategy. We summarize what is known, unknown, and hotly debated regarding the coding and non-coding regions of HEV ORF1, and present a model of how HEV replicates its genome.

## 1. Introduction

Hepatitis E virus (HEV) is a positive-sense, 5′-capped, single-stranded RNA virus of the *Hepeviridae* family [1]. The virus is ~7.2 kB in length and was recently discovered to be quasi-enveloped [2]. The HEV strains that infect humans are classified in the genus *Orthohepevirus*, and are primarily transmitted through contaminated water sources or through the consumption of undercooked, infected meat derived from swine, deer, or wild boar [3].

Acute hepatitis from HEV is generally self-limiting in healthy patients, and symptoms (which include jaundice, nausea, vomiting, and fever) tend to resolve approximately 1 month post-infection [4]. However, two subpopulations of patients notably experience severe clinical outcomes from HEV infection. The first are immunocompromised persons, such as organ transplant recipients on immunosuppressive regimens for whom HEV can lead to chronic hepatitis and rapid development of liver cirrhosis [5]. Secondly, pregnant women in the third trimester experience up to a 30% mortality rate from infection with HEV genotype 1, particularly in Northern India [6]. The underlying mechanisms leading to pregnancy-related pathology are not well understood.

In immunocompromised patients, chronic HEV is first treated by reducing immunosuppressive therapy where applicable (effective in ~70% of patients), and if this is unsuccessful, ribavirin monotherapy is used [7]. Though ribavirin therapy is ~90% effective in clearing infection, it is associated with severe side effects and, more recently, ribavirin-resistant strains have emerged in Western Europe [8,9,10]. Additionally ribavirin is highly teratogenic and cannot be used for the treatment of pregnant women, who suffer disproportionately severe effects from HEV infection and currently have no treatment options other than supportive care [6].

In this review, we focus on the mechanisms of HEV genomic replication, starting from uncoating after the virus has entered the host cytoplasm to the process of asymmetric genomic replication and translation of viral proteins from subgenomes. We also cover, in detail, the previously published literature on open reading frame 1 (ORF1) of HEV which encodes the replicative machinery of the virus, as well as the 5′ non-coding and junctional regions flanking ORF1.

## 2. Genomic Organization of ORF1

**Figure 1:**
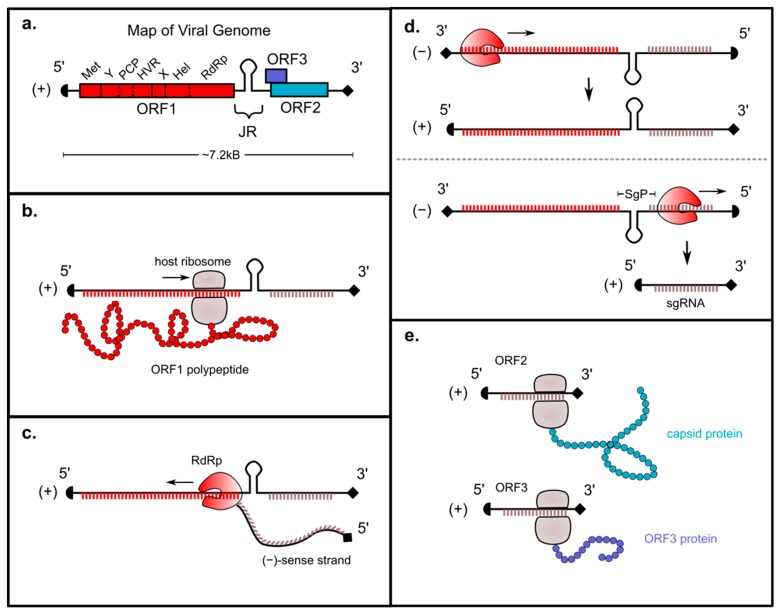
Genomic organization and replicative mechanism of hepatitis E virus. (**a**) Map of HEV genome including domains encoded by ORFs 1–3. (**b**) After viral entry and uncoating, ORF1 is translated by host ribosomes into a polyprotein that includes the RdRp. (**c**) The RdRp transcribes a (−)-sense full-length genome from the (+)-sense strand. (**d**) The (−)-sense genome is a template for transcription of two (+)-sense RNAs: a full-length transcript and a subgenomic (sg) RNA encoding ORFs 2 and 3. The latter is transcribed from the subgenomic promoter (SgP). (**e**) The capsid protein and HEV viroporin are translated from the subgenomic RNA.

HEV is organized into three ORFs. ORF1 is the largest, comprising ~5 kB of the virus and encoding the enzymes required for genomic replication including the methyltransferase (Met), RNA helicase (Hel), a putative papain-like cysteine protease (PCP), and the RNA-dependent RNA polymerase (RdRp) (Figure 1a). The genome additionally contains ‘X’, ‘Y’, and ‘hypervariable (HVR)’ domains whose precise functions are not understood but that are known to play crucial roles in viral replication. One study has also described a fourth open reading frame, ORF4, which is only present in HEV genotype 1 and is translated into a protein that increases activity of the RdRp [11].

## 3. Genomic Replication Strategy

The (+)-sense HEV genome is capped at the 5′ end and polyadenylated at the 3′ end, and can therefore be translated directly by host ribosomes [12]. After viral entry and uncoating, the ORF1 region within HEV is translated into a polyprotein that includes the RdRp (Figure 1b). There is debate over whether this polyprotein is then processed into smaller subunits; a topic that will be discussed in more detail later in this review. The RdRp then transcribes a (−)-sense full-length genome using the (+)-sense strand as a template (Figure 1c) [13]. This (−)-sense genome, which is transcribed in relatively small amounts, serves as a template for transcription of two different (+)-sense RNAs: a full-length (+)-sense transcript that is packaged into progeny virions, and a smaller “subgenomic” (sg) (+)-sense RNA encoding ORFs 2 and 3 (Figure 1d) [13,14]. The capsid protein and HEV viroporin, which are respectively required for viral packaging and release, are translated from the subgenomic RNA (Figure 1e) [14,15,16].

## 4. Roles of ORF1-Encoded Regions in HEV Replication

### 4.1. Methyltransferase

#### 4.1.1. Introduction

After HEV was identified as non-A, non-B hepatitis in 1983, research into the functional domains of the virus began [17]. With the help of bioinformatic analysis and sequence alignments between HEV and other viruses, the sequence homology of several domains emerged, including motifs indicative of a methyltransferase domain [18]. Functional testing of this domain is still ongoing in many aspects, but several key observations about its activity were made early on.

#### 4.1.2. Function

The genomic RNA of HEV is known to be capped, based on binding assays to monoclonal antibodies and competitive binding experiments [19]. The region responsible for capping the genome is encoded by nucleotides 1–979 of HEV ORF1, which is translated into a protein of 110 kDa that possesses methyltransferase and guanylyltransferase activity [20]. This protein allows for the transfer of a methyl group to guanosine triphosphate (GTP), giving m7-GTP, which is then covalently coupled to the 5′ end of the HEV genome. A similar mechanism is employed by other (+)-sense RNA viruses in the ‘*alphavirus*-like’ supergroup of RNA viruses [21,22,23,24,25,26,27].

Though sequence homology with other viruses initially suggested that the methyltransferase domain was located from nucleotides 26 to 240, the experimentally validated domain required for capping stretches over half of ORF1, encompassing the methyltransferase, X domain, putative protease, Y domain, and HVR domains, terminating within the helicase domain [18,20]. Therefore, when annotating the viral genome, there are discrepancies in the literature on the precise endpoint of the methyltransferase domain. Shorter peptides expressed from the HEV genome are not capable of performing the enzymatic functions of a capping enzyme, indicating a likely multifunctionality of the protein product of ORF1 [20]. Finally, this protein product is also tightly membrane-bound; an interaction that persists after treatment with EDTA, high concentrations of salts, or in solution at a pH of 11. Therefore, the ORF1 protein appears to somewhat mimic the behavior of integral membrane proteins [20].

#### 4.1.3. Clinical Relevance

Several studies have shown that patients with acute viral hepatitis (AVH) and acute liver failure (ALF) caused by HEV were infected with virus containing mutations in various domains of ORF1, including in the methyltransferase domain. Mutations in this region can have pro- or antiviral effects. In one study, it was found that the D29N and V27A mutations in the methyltransferase domain of genotype 1 HEV led to an increase in viral load, and were identified in patients experiencing ALF but not in those experiencing the relatively less severe symptoms of AVH (GenBank accession no: AF459438) [28]. Conversely, the single point mutation H105R led to a decrease in viremia, suggesting that this could be a potential therapeutic target in the future [28]. Finally, another clinical study found six amino acid substitutions within ORF1 that were significantly associated with fulminant hepatic failure (FHF) caused by HEV genotype 1; one of which was located within the methyltransferase domain, whereas five were located within the region expressed in the study that identified the capping enzyme activity of ORF1 amino acids 1–979 [18,20]. These amino acid substitutions are as follows: F179S (located in the methyltransferase domain); A317T (located in the putative Y domain); and I676L, T735I, and L736P (all located within the HVR) [29].

Ultimately, though the methyltransferase domain is one of the better understood regions of HEV, questions still remain as to whether the protein is a discrete, functional unit of a processed polyprotein, or if it is one functional region of a large multifunctional protein.

### 4.2. X/Macro and Y Domains

#### 4.2.1. Introduction

The X domain (also known as the macro domain) and Y domains of HEV are, at this time of writing, enigmatic to say the least. As with other domains of HEV ORF1, the functions of the X and Y domains were initially deduced based on sequence alignments with other viruses [18]. Since then, several mechanistic studies have been performed, elucidating the importance of these regions in the viral lifecycle. These two domains will be discussed together due to the dearth of knowledge currently available about either; there is much yet to be gained by interrogating these domains in a rigorous and exhaustive manner.

#### 4.2.2. X Domain Functions

The HEV X domain is also known as the ‘macro domain’ because it exhibits weak sequence homology to human macro domain-containing proteins, which are ADP ribose-binding molecules [30]. Mono and poly-ADP ribosylation are post-translational modifications (PTMs) that occur within bacterial and higher-order cells, and are necessary for a number of biological processes including DNA repair, transcription, chromatin biology, and long-term memory formation [30,31,32]. Interestingly, it was discovered that several viral macro domains, including the X domain of HEV, bind ADP ribose metabolites with differential activity [33]. The HEV X domain binds poly-ADP ribose with substantial affinity and may play a role in viral replication and/or translation. This binding of poly-ADP ribose by the HEV X domain occurs even in the presence of a poly-A competitor, and may be mediated via recruitment of cellular factors to the viral replication complex while bound to RNA [33]. The ADP ribose-binding property of the X domain of HEV remains a rich area for further study.

Other suggested functions of the HEV X domain include a role in viral replication and *cis* interactions with the methyltransferase and ORF3 proteins. The HEV X domain shows high sequence homology to the active site of the coronavirus (CoV) cellular X domain-associated macro domain protein/ADP ribose-1”-monophosphatase (CoV Appr-1”-pase), and mutations in the putative catalytic site of the HEV X domain at N809A, H812L, G816A, G816V, G817A, or G817V severely reduce or completely abrogate viral replicative ability based on experiments using a reporter replicon of the SAR55 strain of genotype 1 HEV (GenBank accession no. AF444002) [34]. A yeast two-hybrid study suggests that the HEV X domain binds the ORF1 methyltransferase at its N-terminus at amino acids 30–90, which includes the methyltransferase catalytic pocket, and that it also binds ORF3 protein at its N-terminal 35 amino acids and C-terminal amino acids 63–123 [35]. Furthermore, the X domain binds both of these regions with its own C-terminal 66 and 67 isoleucine, and 101 and 102 leucine, which are conserved residues across all HEV genotypes [35]. Interestingly, it was found that the methyltransferase and the ORF3 protein bind the macro domain competitively, which the authors suggest is a regulatory mechanism as the virus likely needs more methyltransferase activity early in infection, as well as the ORF3 protein, which is a multifunctional phosphoprotein with ion channel activity necessary viral egress later in infection [15,35].

#### 4.2.3. Y Domain Function

To date, there is very little known about the Y domain of HEV outside of the fact that it possesses sequence conservation of several motifs within this region across all known HEV genotypes, and shares sequence homology across several motifs with other known viruses. Specifically, in silico analysis suggests a putative palmitoylation site at residues C336–C337, and an alpha helix segment stretching from residues L410 to E416 (both motifs conserved across known HEV genotypes) that is predicted to bind the cytoplasmic membrane [36]. Additionally, mutating conserved residues C336A, C337A, and W413A within the Y domain of genotype 1 HEV causes viral non-viability in HepG2C3A human hepatoma cells [36]. These motifs are thought to lie within predicted RNA stem loops, and non-viability is thought to occur due to a disruption in the RNA secondary structure [36]. Furthermore, sequence analyses of over 50 genera of viruses demonstrate that methyltransferase and guanylyltransferase activities of an encoded capping enzyme possess a conserved core region which is followed by an extended region known as the ‘iceberg region’. This iceberg region, whose secondary structure (but not sequence) is highly conserved, contains putative membrane binding association sites that contribute to the assembly of viral replication factories in other better-studied viruses [37]. In this instance, the extended minimal enzymatically active region of the HEV methyltransferase domain is predicted to include the entirety of the Y domain of HEV [37], but it has not yet been shown if the loss of viral viability due to mutations in this region is due to loss of methyltransferase activity. While these findings increase our current knowledge of the Y domain, there is still much to learn about the function of this domain and understanding why it is critical for viral replication.

#### 4.2.4. Clinical Relevance

Not much is known about the clinical relevance of the X and Y domains of HEV, but a few studies have thus far shed some light on their importance in pathology. One such study analyzed HEV quasispecies in two groups of solid organ transplant patients; one of these groups went on to establish chronicity and the other group cleared the infection. Between these two groups, there was greater sequence heterogeneity in the HVR region of HEV and within the X domain in the group that developed chronic infections when compared to the group who cleared in infection, suggesting a role in the severity of pathogenesis dictated by the composition of these regions [38].

There is also some evidence suggesting the involvement of the X domain in cellular iron transport. Based on studies using a yeast two-hybrid system, the HEV macro domain protein interacts with the light chain of human ferritin [39]. Furthermore, HUH7 human hepatoma cells overexpressing the HEV macro domain protein exhibit lower levels of secreted ferritin when compared to naive cells, despite cellular iron metabolism/homeostasis and levels of transferrin receptor 1 or ferroportin remaining unaffected [39]. The relevance of these findings in the context of pathogenesis of HEV is still under investigation.

Once again, the presence of macro domains is conserved across the alpha-like supergroup of positive sense RNA viruses, including coronaviruses, rubella virus, HEV, and mouse hepatitis virus (MHV). MHV, a coronavirus, induces high levels of inflammatory cytokines and is known to induce acute viral hepatitis in mice. Strikingly, a recombinant MHV strain A59 with a single amino acid substitution within the macro domain enzymatic core N1348A, encoded by the virus, not only led to slightly reduced titers when introduced to mice, but it did not induce liver disease and induced much lower levels of the inflammatory cytokine IL-6 [40]. While this virus is a distant relative of HEV, these findings may lead to further study of this domain in the context of human pathogenesis, hopefully shedding more light into this enigmatic viral region.

### 4.3. Putative Protease Region

#### 4.3.1. Introduction

The putative protease region of HEV and its function(s) are still under debate. The region was first identified as a putative protease based on sequence alignment with rubella virus and, since then, it has been studied in a variety of expression paradigms, leading to directly conflicting results. As it stands, there are two major hypotheses in the HEV field regarding this domain: (1) that this region acts as a true virally encoded protease that processes the HEV polyprotein into discrete functional units; or (2) that this region does not process the viral polyprotein, and if it does harbor proteolytic activity, the targets must be cellular and not viral. The studies supporting each hypothesis are outlined below.

#### 4.3.2. Evidence that the Putative Protease Region is Responsible for Processing the HEV Polyprotein into Discrete Function Units

Several studies suggest that the polyprotein of HEV is processed but disagree on the class of protease that is encoded by this region. These studies are summarized in this section.

1. Evidence for a cysteine protease that processes ORF1 into nine protein subunits: In silico analysis predicted a papain-like beta barrel fold and identified the proteolytic dyad of this putative papain-like cysteine protease (PCP) as residues C434 and H443 [41]. A putative zinc-binding motif and three potential disulfide bridges were also predicted in the PCP region [41]. To experimentally characterize this region, one group used a baculovirus expression system to overcome the low yield of ORF1 protein expressed in cells during natural infection. Using matrix-assisted laser desorption ionization time of flight mass spectrometry (MALDI-TOF), they observed nine distinct protein species of ORF1 of genotype 1 of HEV appearing in a time-dependent manner upon expression in non-natural host insect cells [42]. Additionally, they observed that treatment with a cysteine protease inhibitor blocked this processing of the ORF1 polyprotein [42].
2. Evidence for processing of ORF1 into two subunits: Another study utilizing a GFP reporter replicon of the genotype 1 SAR55 strain of HEV showed that site-directed mutagenesis of the following residues in the PCP domain abrogated GFP expression in HUH 7 S10-3 cells, used in the study as a proxy for viral replication: C457A, C459A, C471A, C472A, C481A, C483A, H443L, H497L, and H590L [43]. This study also found that expressing histidine and HA-tagged replicons in HUH7 S10-3 cells showed the 186 kDa protein processed into approximately 35 and 78 kDa fragments.
3. Evidence for chymotrypsin-like activity of the putative protease: One study found that purified and dialyzed fragments of HEV ORF1 and ORF2 were processed into smaller fragments when incubated with purified protein from the PCP region spanning amino acids 440–610, and that inhibitors to chymotrypsin halted this processing, suggesting that this region harbors a different class of proteolytic activity [44].
4. Evidence for serine protease cleavage sites within the PCP region: Conversely, a recent study found two conserved putative cleavage sites for cellular thrombin and one putative cleavage site for cellular factor Xa within HEV ORF1, conserved across all HEV genotypes; the factor Xa site was suggested within the PCP domain at amino acid 560, and the thrombin sites in X domain (between 846 and 862 depending on HEV genotype) and in RNA-dependent RNA polymerase (between 1218 and 1235 depending on genotype) [45]. To characterize these sites, the authors made mutations in an HEV genotype 1 reporter replicon (GenBank accession no: AF444002.1) and found viral replication to be impeded, and that treating HUH7 S10-3 cells with a serine protease inhibitor or siRNA to interfere with these cellular factors also inhibited viral replication [45].

#### 4.3.3. Evidence against Processing

While the studies listed above implicate the polyprotein as being processed over the course of the HEV replication cycle, few utilize biologically relevant systems that accurately mimic HEV infection, and none were conducted in the context of a natural infection cycle. Conversely, studies looking at ORF1 protein function and localization did not find evidence of polyprotein processing. One such study concluded that in vitro translation of ORF1 protein did not harbor intrinsic proteolytic activity when the protein was purified; subsequent in vitro expression of proteins in HeLa or HUH7 and pulse-chase experiments with radioactive methionine did not show processing, and suggest that the ORF1 polyprotein localizes to endoplasmic reticulum membranes [46]. Other studies expressing epitope-tagged ORF1 protein of genotype 1 and genotype 3 of HEV in 293T cells did not show processed protein via Western blot [47], nor did IP of tagged ORF1 show processing in the human hepatoma line HepG2 [48]. Additionally, HEV expressed via a vaccinia virus system did show processed 107 and 78 kDa products, however, mutating the putative catalytic residue C483 failed to abolish this cleavage, leading the authors to express doubts regarding the presence of a functional PCP within ORF1 [49]. Another group investigating the innate immune evasion strategies of this region of ORF1 (a topic beyond the scope of this review), found deubiquitination activities associated with the PCP region, and during the course of the study, expressed truncated versions of ORF1 containing the PCP and were unable to observe any cleavage products [50].

Any study relying on (over)expression of the PCP, or any ORF1 subunit for that matter, will ultimately have to prove that such domain taken outside the context of the ORF1 polyprotein is still functioning properly. Otherwise, the relevance of any data will be very difficult to determine. Ultimately, the function of the putative protease domain is still as enigmatic as ever, and future studies will hopefully be able to shed more unambiguous light on the functions of this region of the HEV replicase.

### 4.4. Hypervariable Region

#### 4.4.1. Introduction

The hypervariable region (HVR) of HEV is located in ORF1 directly downstream of the putative papain-like cysteine protease (PCP) domain. It is bound by the conserved sequences TLYTRTWS and RRLLXTYPDG at the N- and C- termini respectively, however, the intervening sequence contains the most divergence of any region in the virus, earning it the name ‘hypervariable region’ [51]. Although no satisfactory alignment can be made between HVRs of different genotypes, the HVRs from all HEV genotypes share the characteristic of being rich in proline [52]. Accordingly, the HVR is also known as the polyproline region.

Amino acid sequence identity of the HVR differs up to 71% across HEV genotypes. Intra-genotypically, it differs up to 31% among genotype 1 isolates, 41% among genotype 3 isolates, 46% among genotype 4 isolates, and 30% between the only two available avian HEV isolates [53]. The HVR is also the primary region accounting for size differences in HEV genomes from different genotypes, since its length can vary depending on the presence of insertions and/or deletions. The HVR was initially thought to span 105 amino acids, however, as additional HEV sequences became available, it was found that the first 35 amino acids of the originally demarcated HVR were not, in fact, hypervariable. Therefore, it is now established that the HVR is typically 70–72 amino acids in length for genotype 1 HEV, 68 amino acids for genotype 2 HEV, 80–86 amino acids for genotype 3 HEV, 84 aa for all genotype 4 HEVs, and 84 aa for avian HEV [53].

#### 4.4.2. Function

The most commonly proposed function for the HEV HVR is that it plays a structural role as a flexible hinge between adjoining ORF1 regions [18]. This is supported by evidence that the HVR overlaps an intrinsically disordered region (IDR); i.e., a protein sequence lacking a fixed three-dimensional structure [51]. IDRs usually have a high proportion of polar and charged amino acids, and structure-breaking amino acids like proline and glycine. The low content of bulky hydrophobic amino acids and high fractions of alanine, glycine, proline, and serine in the HEV HVR across genotypes is consistent with the properties of known IDRs. Therefore, the HVR may be involved in mediating conformational changes that affect protein–protein interactions [51].

There is also evidence suggesting that the HVR influences host tropism. HVR sequence divergence is >2-fold greater for HEV zoonotic genotypes 3 and 4 than for human genotype 1, suggesting a potential association between sequence heterogeneity and the number of hosts [51]. Furthermore, in vitro studies reveal that insertion of sequences in the HVR can expand the host range of HEV strains in vitro (see Insertions) [54].

Finally, the HVR is known to be essential for viral replication and, despite its great sequence heterogeneity, it exerts this effect in a genotype-specific manner. Deletions in the HVR or swapping HVR sequences between strains of HEV from different genotypes both result in major reductions in viral replication efficiency [55]. The specific role of the HVR in viral replication remains to be elucidated.

#### 4.4.3. Insertions

Multiple analyses of HEV isolates from chronically infected patients have revealed that the HVR can acquire insertions over time, either from other regions of the viral genome, or from human genes [56,57]. It is unclear how and why these insertions arise, however, two HEV strains containing human ribosomal insertions in the HVR have notably been found to harbor increased replicative capacity in cell culture. These genotype 3 strains, named LBPR-0379 and Kernow-C1, respectively acquired insertions in their HVR regions from the S19 and S17 ribosomal protein genes [58,59]. Interestingly, though the S19 and S17 inserts are both derived from highly conserved ribosomal protein gene sequences, they differ considerably from one another. In both cases, the strains with ribosomal inserts constituted minor quasispecies of the original clinical isolates from feces of chronically infected patients. Upon repeated passaging in vitro, the strains with insertions became the major species, indicating that these insertions conferred a replicative advantage in cell culture. Indeed, the Kernow-C1-p6 strain (“p6” indicating six passages in cell culture) is one of the most commonly used HEV strains in cell culture studies due to its robust replicative capacity. In addition to increased replicative capacity in cell culture, the S17 insertion has also been shown to expand the host range of several HEV strains in vitro, suggesting a potential role in host adaptation [59]. Finally, the S17 insertion contains a nuclear localization sequence leading to nuclear import of the ORF1 protein, however, the significance of this change in subcellular localization is not clear and remains a topic for further study [60]. Much remains to be understood about the HEV HVR and the effect of insertions in this region.

### 4.5. Helicase

#### 4.5.1. Introduction

Helicases are enzymatically capable of unwinding RNA duplex structures, a process that is coupled with NTP hydrolysis and responsible for the observed NTPase activity in most, if not all, of the helicases described to date (reviewed in [61]). RNA helicases fall under three superfamilies, with each helicase being assigned a family based on sequence similarity to conserved motifs and putative helicase and NTPase activity. All three superfamilies share two motifs, known as Walker A and Walker B sites (first characterized by Walker et al., 1982, *EMBO*), which make up an NTP-binding motif. The A site contains a run of hydrophobic residues followed by a conserved GxxxxGKS/T site (where x represents any amino acid), and the B site begins with a run of hydrophobic amino acids followed by an asparagine. The Walker A site is thought to be involved in directly binding beta and gamma phosphates of NTPs, while the B site chelates Mg^2+^ of the Mg–NTP complex. The HEV helicase falls under superfamily 1, which possesses seven conserved motifs arranged in a co-linear fashion (segments I, Ia, II, III, IV, V, and VI). Motif VI is thought to bind nucleic acids due to the high number of basic residues, namely arginine [61].

#### 4.5.2. Function

Multiple studies have been published in the past several decades aimed at mechanistically understanding and mapping the function of the HEV helicase. Enzyme-linked immunosorbent assays (ELISAs) have shown that the HEV helicase possesses NTPase activity and is capable of hydrolyzing ATP [62]. Deletion of domain I or domain IV results in an increase in NTPase activity, deletion of domain II reduces NTPase activity but does not affect RNA unwinding activity, and deleting domains Ia or domain III severely decrease both NTPase and unwinding activity [62]. Furthermore, the protein fragment from amino acids 960 to 1204 of the helicase domain unwinds RNA duplexes with 5′ overhangs exhibiting a 5′–3′ polarity, and mutating helicase domain motif I (GKS-GAS) or motif II (DEAP-DAAP) abrogates RNA duplex unwinding activity and diminished ATPase activity [63].

Further, a study utilizing a radioactive phosphate assay revealed that the aforementioned HEV helicase domain ranging from amino acids 960 to 1204 from genotype 1 HEV exhibits 5′ NTPase activity, suggesting that this activity is essential during RNA cap formation [64]. The same group purified the protein from the aforementioned region of ORF 1 (amino acids 960–1204) after expression in *Escherichia coli* and found that this protein hydrolyzes all rNTPs efficiently, but exhibits a preference for dATP and dCTP [63].

Other mutations made in the helicase domain of HEV genotype 3 have varying effects on viral replication in a reporter replicon system expressing *Renilla* luciferase. For example, the A1213V and V1213A point mutations respectively reduce replication of the genotype 1 SAR55 and genotype 3 SHEV replicon strains. Additionally, the S605P mutation within the X domain combined with an I978V mutation in the helicase domain have a cumulative negative effect on viral replication [65].

#### 4.5.3. Clinical Relevance

In patients with fulminant hepatic failure caused by genotype 1 HEV, it is common to see the following mutations in the helicase region: L1110F and V1120I [66]. These mutations slightly decrease ATPase activity in the helicase domain but do not alter RNA duplex unwinding [66]; mutations L1110F and V1120I within the helicase domain from clinical isolates were found to impart a higher incidence of fulminant hepatic failure (FHF) in patients when compared to patients who experienced acute viral hepatitis (AVH) [29].

Additionally, a study aimed at finding novel inhibitors to this virally encoded protein domain via virtual screening demonstrated that the saturation mutagenesis producing synonymous mutations within the helicase domain did not affect in vitro-transcribed RNA synthesis, suggesting non-conservation of the genome, and that the nucleotide sequences therein are dispensable at the transcriptional level [67].

### 4.6. RNA-Dependent RNA Polymerase

#### 4.6.1. Introduction

As with most RNA viruses (retroviruses excluded), HEV encodes an RdRp that synthesizes sense and antisense RNA transcripts using antisense and sense viral RNA templates, respectively. In the Burma reference strain of HEV, the RdRp spans nucleotides 1249–1671 (GenBank accession M73218), and it is located downstream of the RNA helicase gene across all genotypes. The HEV RdRp is phylogenetically classified in supergroup III, along with the RdRps from rubella virus and beet necrotic yellow vein virus [68]. Unfortunately, there are no structures available for any supergroup III RdRps, including the HEV RdRp [69]. Like RdRps of many other (+)-strand RNA viruses, the HEV RdRp contains a highly conserved GDD motif constituting its catalytic triad. Point mutations in the GDD motif abolish HEV replicative activity [68].

HEV employs an asymmetric genomic replication mechanism which is thought to occur in the endoplasmic reticulum. The sense viral RNA is first transcribed into an antisense intermediate RNA strand which is subsequently used as a template to transcribe both a full-length sense RNA genome to be packaged into progeny virions as well as a shorter subgenomic RNA (sgRNA) that is used as a template to translate the ORF2 and ORF3 proteins. The HEV RdRp binds to the 3′ UTR of the sense strand to produce the antisense intermediate strand and to the 5′ UTR of the antisense strand to produce the full-length sense RNA genome. The RdRp also binds to a subgenomic promoter (SgP) spanning a 44 nt region from the 3′ end of the ORF1 to the transcriptional start site (TSS) of ORF3 [14,70] and a 9 nt genetic element from the TSS to the translational start site of ORF3, thus extending beyond the junction region which has been previously implicated in the transcriptional control of HEV subgenomic RNA [71]. It is not known how the RdRp regulates binding to these multiple promoter regions, however, in vitro studies testing the binding affinity of purified RdRp protein have shown that RdRp has the highest affinity for the 3′ UTR of the sense strand, than for the 5′ UTR of the antisense strand, and relatively lower affinity for SgP in the antisense strand [72]. Much remains to be understood about the kinetics of transcription of these different RNA species by the HEV RdRp during viral replication.

#### 4.6.2. Host and Viral Protein Interactions

Viral RdRps often interact with other viral or host proteins that can complement RdRp activity, serve as scaffolds for the viral replicase complex, or modulate RdRp activity to influence levels of transcription. Yeast two-hybrid screening has shown that the RdRp self-interacts, a characteristic shared by many (+)-sense strand RNA viruses such as poliovirus and hepatitis C virus; however, it is not known whether these interactions amount to dimer formation or higher-order oligomers [73,74,75]. The HEV RdRp has also been shown to interact with the HEV PCP, and it likely interacts with the methyltransferase and helicase domains since these exert complementary functions to RNA transcription [73].

The HEV RdRp has also been shown to interact with several host and immune factors. It binds the host interferon-induced protein IFIT1, with the proposed function that it sequesters IFIT1 to inhibit its anti-translational activity [76]. RdRps from HEV genotype 1 contain a highly conserved miR-122 target site, and in vitro studies show that HEV strains from genotypes 1 and 3 exhibit increased replication rates in the presence of miR-122 overexpression, as well as decreased replication when miR-122 is knocked out [77]. Therefore, miR-122 may be a proviral factor for HEV as it is for hepatitis C virus (HCV), though the specific role of miR-122 in the context of the HEV lifecycle is not known [77,78]. Finally, a curious feature of the RdRp is that it contains multiple B cell epitopes (based on analysis of genotype 1 strains from patient isolates), though it is not clear why an antibody response would target the RdRp [79].

#### 4.6.3. Clinical Relevance and Therapeutic Potential

Several mutations in the RdRp region have been correlated with adverse clinical outcomes. A study in North India analyzing clinical isolates from pregnant patients with genotype 1 HEV infection found that C1483W and N1530T point mutations in the RdRp were correlated with high viral loads and poor clinical outcomes (Reference strain: GenBank ID AF459438) [8]. Similarly, in immunocompromised patients suffering chronic infections with genotype 3 HEV, a G1634R point mutation in the RdRp (Reference strain: GenBank ID NP_056779) was correlated with higher plasma HEV RNA levels and initial resistance to ribavirin treatment (however, additional rounds of treatment or prolonged treatment were effective) [9]. In vitro studies of the G1634R point mutation have shown that it confers a replicative advantage to HEV strains in cell culture systems, mirroring the higher viral loads seen in patients, and providing an explanation for the reduced efficacy of ribavirin [9,10]. Additional genotype 3 mutations in the RdRp have been identified in patients showing clinical resistance to ribavirin: Y1320H, G1634K, K1398R, V1479I, and Y1587F; however, in vitro studies of these mutations did not demonstrate increased replication fitness or resistance to ribavirin [80]. Further work is needed to fully understand the clinical implications of mutations in the RdRp, however, these findings already suggest that RdRp sequencing may be a useful prognostic tool for patients and clinicians.

The RdRp is also an attractive therapeutic target. Ribavirin, the only existing treatment for chronic HEV infection, is a nucleoside analog that inhibits RdRp activity and is highly effective in certain patient subpopulations [81]. In vitro studies also suggest that zinc salts and RNAi can effectively inhibit the HEV RdRp [82,83].

## 5. *cis*-Acting Regulatory Elements

The HEV genome contains two known *cis*-acting regulatory elements (CREs) that play roles during genomic replication: the 3′ non-coding region (NCR) and the subgenomic promoter (SgP). Both the 3′ NCR and the SgP likely contain secondary structures and sequences that are highly conserved across diverse HEV genotypes [71,84]. These regions also bear similarities to the corresponding sequences in alphaviruses such as Sindbis virus and Semliki Forest virus, which employ an asymmetric genomic replication mechanism like HEV [85,86]. A number of studies focused on characterizing and fine-mapping these regions have added substantial knowledge to our understanding of HEV replicative mechanisms. Here, we review what is known about the HEV CREs and briefly discuss the remaining knowledge gaps.

HEV, which contains a (+)-sense single-stranded RNA genome, requires transcription of a complementary antisense template RNA as a key step in genomic replication. Initiation of antisense RNA transcription requires binding of the RdRp or replicase complex to the 3′ end of the sense strand; therefore, this region plays a critical and early role in replication of the viral genome. Studies have confirmed that two stem loop structures located at nucleotides 7089–7163 and 7173–7194 (Ref: AF076239) as well as the 3′ poly-A tail are critical for interaction with the RdRp, and that the two stem loops are conserved across diverse HEV genotypes [84]. Mutations in the stem loop regions or deletion of the poly-A tail result in greatly reduced binding of the RdRp. Another set of studies generating chimeric HEV strains have also shown that the 3′ NCR can be swapped between the Sar55 (GT 1, GenBank accession no. AF444002), Mex-14 (GT 2, GenBank accession no. M74506), and Meng (GT 3, GenBank accession no. AF082843) strains of HEV without a resulting decrease in replication levels, despite sequence differences (the Sar55 3′ NCR shares 81% and 74% nucleotide identity with Mex-14 and Meng strains, respectively) [87]. These results collectively suggest that the RdRp recognizes structural motifs in the 3′ NCR and is not as sensitive to sequence variations when binding to initiate antisense RNA synthesis.

The subgenomic promoter (SgP) is a conserved sequence located at nucleotides 5080–5132 (Ref: Burma M73218) which, on the antisense template RNA, is critical for synthesis of the (+)-sense subgenomic RNA (sgRNA) [71,88,89]. The SgP spans a portion of the ORF1 3′ coding region (5080–5109), the junction region between ORF1 and the transcriptional start site (TSS) of ORF3 (5110–5123), and a 9 nt genetic element from the TSS to the translational start site of ORF3 (5124–5132) (Figure 1d). The SgP is required for RdRp-mediated transcription of the capped, ~2200 bp bicistronic sgRNA encoding ORF2 and ORF3, and mutations in the SgP accordingly abolish ORF2 and ORF3 protein translation [90]. A highly conserved stem loop structure is located at nucleotides 5080–5123 (Ref: Burma M73218), and mutations in the stem loop—even those that preserve the stem loop structure—lead to a decrease in viral replication, suggesting that both the sequence as well as the secondary structure are critical [71]. The SgP sequence is not present in the 3′ NCR of the sense genome, suggesting that the RdRp or replicase complex employ a separate mechanism for binding to the 3′ NCR.

Electrophoretic mobility shift assays have shown that the RdRp exhibits a higher binding affinity to the 3′ NCR than SgP, however, further studies are required to fully understand how RdRp interactions with these target sites are regulated [84]. Unfortunately, due to inefficient cell culture systems and detection assays, the kinetics and relative levels of antisense, full-length (+)-sense, and sgRNA production have not been measured; these data will provide important insight into the roles of the CREs during the viral lifecycle.

## 6. Conclusions

HEV is a globally prevalent pathogen causing a heavy clinical burden in diverse geographical and patient populations. Since the first infectious clone for HEV was created in 1991 [91], major advancements have been made in better understanding replicative mechanisms of the virus. However, many large knowledge gaps remain regarding specific functional roles of entire viral domains and, additionally, there is conflicting data in the literature that requires resolution (Box 1). As global awareness of HEV grows and tools and models to study the virus improve, we anticipate that key knowledge gaps will be bridged and provide a foundation for the development of effective therapies against this dangerous and enigmatic pathogen.

Box 1 **Open Questions.**

1. Which host factors are essential for HEV RNA replication?
2. Does HEV replication lead to membrane rearrangements similar to other (+) RNA viruses?
3. Does ORF1 polyprotein get processed? If so, how?
4. Does the PCP region contain protease activity? If so, against what targets?
5. What is the role of the HVR? How do insertions in this region confer cell culture adaptation?
6. What is the role of the Y domain in the HEV replication cycle?
7. What is the structure of the ORF1 polyprotein, or structures of ORF1-encoded proteins (if processed)?
8. What are the kinetics and levels of transcription of antisense, full-length (+)-sense, and sgRNAs; and what are the mechanisms regulating expression of these elements?
9. What are the components of the replicase complex for HEV? Does the RdRp self-oligomerize?
